# Working at home and alcohol use

**DOI:** 10.1016/j.abrep.2021.100377

**Published:** 2021-09-16

**Authors:** Morten Birkeland Nielsen, Jan Olav Christensen, Stein Knardahl

**Affiliations:** National Institute of Occupational Health

**Keywords:** Telework, Telecommuting, Flexible work arrangements, Substance use, Drinking

## Abstract

Working at home has become a frequent work arrangement following the COVID-19 pandemic. However, little is known about how working at home influence alcohol use among employees. This study examines associations between working at home at least 15 h per week and alcohol consumption using data from a *pre-COVID-19* sample. Self-reported questionnaire data on working at home and alcohol use from a large cross-sectional sample of Norwegian employees (N = 14,728). Data were collected between 2004 and 2019 and were analyzed by ordinal logistic regressions. Working at home for >15 h per week was significantly associated with alcohol use (OR 1.67, 95% CI: 1.30 – 2.16). The association remained significant after adjusting for age, gender, leadership position, and educational level. Working at home may facilitate alcohol use that otherwise would not happen. Organizations must ensure that policies and procedures are in place to prevent alcohol use during working hours among employees working at home.

## Highlights

• The use of own home as a workplace is increasing.
• Working at home is associated with higher alcohol consumption.
• Working at home may facilitate alcohol use that otherwise would not happen.

## 1 Working at home and alcohol use

Following the outbreak of the COVID-19 pandemic in 2020, many office workers were forced to self-isolate and work at home to reduce the risk of virus transmission. Although this use of home office work initially was considered as a temporary measure to decrease infection rates, it now seems that these changes in work arrangements following COVID-19 have accelerated a trend where telecommuting and working at home are likely to continue also after the pandemic (Spurk & Straub, 2020). Despite the growing importance and widely spreading practice of working at home, it is unknown whether this kind of work arrangement is good or bad for employees (Gajendran et al., 2007, Li et al., 2020). There are also major knowledge gaps in the literature on telecommuting. Existing studies have mainly focused on work related factors such as work-family imbalance, job satisfaction, performance, turnover intent, and role stress (Gajendran et al., 2007), whereas less is known about how working at home influences lifestyle factors such as physical activity, eating habits, and alcohol use.

Regarding alcohol use, a substantial proportion of Norwegians changed their drinking behavior following the Covid-19 pandemic (Bramness et al., 2021). Quantifications of changes in alcohol consumption show that the upper 5 to 10% of those who consumed alcohol increased their consumption and hence the prevalence of heavy drinking increased (Rossow et al., 2021). Another study from the same sample showed that female gender and younger age were risk factors for both less and more drinking, and the increase in alcohol consumption was stronger with higher educational level (Bramness et al., 2021). Findings from the US show that 1 in 3 Americans were more likely to drink alcohol during working hours while in lockdown (American Addiction Centers, 2021). Considering these changes in alcohol consumption coincided with the increased use of working at home arrangements, it is reasonable to question whether the use of home office is a potential risk factor for higher alcohol consumption (Monteiro et al., 2020). To answer this question, this study examined associations between working at home and alcohol consumption using data from a *pre-COVID-19* sample to provide information that pertains to a “normal situation”. As working at home has been proposed as a risk factor for engaging in counterproductive work behavior (Holland et al., 2016), we expect a positive association between number of hours working at home and higher levels of alcohol use.

## 2 Methods

### 2.1 Procedure and sample

This study was based on data from “The New Workplace: Work, Health and Participation in Working Life” project at the National Institute of Occupational Health (NIOH) which is a survey of Norwegian employees working in a full time or part time position (Christensen and Knardahl, 2010, Finne et al., 2014). In accordance with the requirements for health research in Norway, this project was approved by the Regional Committees for Medical and Health Research Ethics (REC) in Norway, has permission from the Data Inspectorate of Norway and was conducted in accordance with the World Medical Association Declaration of Helsinki. Subjects were recruited from organizations based in Norway that were contacted and offered participation, or that requested participation after obtaining information about the ongoing study disseminated by NIOH on their website. A variety of job types and organizations, including municipalities, insurance companies, health institutions, and public organizations, were represented in the survey. After excluding workers that were on absence, all employees were mailed a letter with information about the survey, informed consent, and ethical considerations. All study participants provided their informed consent. Responses were treated anonymously in analyses. Data were gathered between 2004 and 2019.

Of the 32,793 employees that were invited to the baseline assessment, 16,442 responded (response rate: 50%). After removing respondents with missing data on the study variables, the final sample comprised 14,728 respondents. Mean age was 43.56 (SD = 10.73) years (range:18–73). The sample consisted of more women (53.8%) than men (45%). In total, 3.4 percent had between 1 and 9 years of education, 33.2% had between 10 and 12 years, 44.8% had between 13 and 16 years, and 18.6% had 16 years or more. Ninety-one percent had a regular full-time employment, and 79% had a day work schedule. Altogether 21% had a leadership position that included personnel responsibility for subordinates.

### 2.2 Instruments

Working at home was assessed with a single item question. After stating “Many employees may work at home, either by bringing work physically to their home or electronically via the Internet (teleworking)”, respondents were asked “How many hours did you spend working in your own home, last week?”. Response categories were “0 h”, “0–2 h”, “2–5 h”, “5–15 h”, and “More than 15 h”. In the current study, we set more than 15 h per week as a cut-off criterion for teleworking. As a regular workday in Norway is 7.5 h, 15 h corresponds to two full days of telework.

In line with a previous study (Nielsen et al., 2015), alcohol use was measured with a single item asking, “How many units of alcohol do you consume in a typical week (1 unit of alcohol is 10–15 g ethanol, i.e., half a liter of (pilsener) beer, 1 glass of red wine, 1 ordinary drink, etc.)?”. Response categories were “0”, “1–2”, “3–4”, “5–6”, “7–9” and “greater than10”. The two latter categories were combined in this study.

### 2.3 Statistical analyses

Ordinal logistic regressions in SPSS 25.0 were conducted to estimate the association between working at home and alcohol use. Cumulative odds ratios (ORs) were calculated to estimate effects. Cumulative ORs estimate the odds of an ordered categorical outcome variable being one category higher versus lower, assuming this ratio is the same for all cut points of the scale. With a categorical predictor, odds for all levels of the predictor are compared with odds for the reference category. Age and gender were included as control variables in all analyses.

## 3 Results

Descriptive statistics for the study variables are presented in Table 1. In total, 1.9 percent of the respondents worked at home for more than 15 h per week. As for alcohol use on a weekly basis, 29.3 percent of the total sample were abstainers, 32.3 percent drank 1–2 units, 19.4 percent drank 3–4 units, 10.8 percent drank 5–6 units, whereas 8.2 percent drank seven units or more. The distribution of alcohol use differed between regular office workers and those working at home greater than 15 h per week (X2 = 27.68; df = 4; p < .001). The prevalence rates indicated a higher alcohol consumption among respondents working at home greater than 15 h per week. Alcohol consumption was not associated with survey year (r = 0.01; p > .05), thus indicating that levels of alcohol use was stable across the 15-year long data collection.

**Table 1:** Descriptive statistics for study variables.

**Variable**	**Categories**	**N**	**%**
Work-at-home >15 h per week			
	No	14,055	98.4
	Yes	223	1.6
Gender			
	Female	7544	53.8
	Male	6472	46.2
Education			
	< 9 years	353	3.4
	10–12 years	3491	33.2
	13–16 years	4711	44.8
	16 years<	1959	18.6
Leadership position			
	No	8827	79
	Yes	2348	21
Alcohol use			
	0	3602	28.3
	1–2	4166	32.7
	3–4	2481	19.5
	5–6	1405	11.0
	7 or more	1071	8.4

Ordinal logistic regression analyses were conducted to further determine differences in alcohol consumption. The findings are displayed in Table 2. Alcohol use was regressed on the predictor variables in three different models. In Model 1, working at home was statistically significantly associated with alcohol use (OR 1.67, 95% CI: 1.30 – 2.16). The association remained statistically significant after adjusting for age and gender in Model 2 and leadership position and educational level in Model 3. The final, fully adjusted model showed that employees working at home for more than 15 h per week had an odds ratio of 1.42 for higher alcohol use. Male gender (OR 1.79, 95% CI: 1.66 – 1.94), higher age (OR 1.02, 95% CI: 1.01 – 1.02), having a leadership position (OR 1.18, 95% CI: 1.07 – 1.30), and higher educational level (OR 1.42, 95% CI: 1.31 – 1.54) were also significantly associated with alcohol use.

**Table 2:** Association between working at home and alcohol use (Ordinal regression).

	Model 1		Model 2		Model 3	
	OR	95% CI	OR	95% CI	OR	95% CI
Working at home	1.67***	1.30 – 2.16	1.45**	1.17 – 1.95	1.42*	1.05 – 1.92
Age	–	–	1.02***	1.01–1.02	1.02***	1.01 – 1.02
Gender	–	–	1.87***	1.76 – 2.00	1.79***	1.66 – 1.94
Leadership position	–	–	–	–	1.18**	1.07 – 1.30
Educational level	–	–	–	–	1.42***	1.31 – 1.54

*p < .05; **p < .01; ***p < .001.

Reference categories: Working at home – On site office work; Gender – female; Leadership position – Non-leader; Education – Low levels.

## 4 Discussion

In a large and diverse sample of employees in Norway, working at home for more than 15 h per week, equaling two full working days, was associated with higher alcohol consumption. The association remained consistent even after adjusting for age, gender, leadership responsibility, and educational level. All of the latter are potential risk factors for alcohol use (Bramness et al., 2021), as was also confirmed by the present analyses. As the data was collected before the COVID-19 outbreak in 2020, the findings reflect a “normal” working situation and the differences in alcohol use cannot be attributed to factors related to the pandemic, including worrying, anxiety, self-medication, and so on.

There are two main causal explanations for why working at home is associated with higher alcohol consumption. First, it may be that that employees with a high consumption choose to work at home to conceal their alcohol use for the employer. Second, working at home reduces the threshold for drinking alcohol and thereby facilitate alcohol use that otherwise would not happen. Common for both explanations is that due to decreased direct supervision from the employer, working at home offers employees more opportunity to engage in nonwork activities and counterproductive work behavior during prespecified work times (Holland et al., 2016). At the office, it is easier to spot signs of alcohol use, such as smell, glassy eyes, slurred speech, performance issues, and frequent tardiness. Without physical contact, it will be more difficult to spot these signs. Consequently, the risk of work-related penalties following alcohol use is perceived as low, at least when compared to on-site work, and that this assessment of risk increases the likelihood for alcohol use.

Working at home reduces face-to-face contact and interaction with colleagues and thereby the opportunity for work-related social support. Social support is an important coping strategy. Research shows that having effective social support is one of the most significant correlates of well-being and is assumed to positively impact health and guard against distress (Dirkzwager et al., 2003). Hence, another explanation for our findings is that working at home reduce the likelihood for work related social support and thereby facilitates the use of other, and more dysfunctional, coping strategies, such as alcohol use, to alleviate distress.

A limitation of the current study is that it did not include information about when the respondents consume alcohol. Hence, we do not know if the alcohol is consumed during or after the workday. Still, as employees working at home seems to have a lower threshold for drinking, a practical implication of our findings is that employers must ensure that policies and procedures are in place to prevent alcohol use during working hours, even when working at home. This includes the training of managers and HR-personnel with regard to identifying signs of potential alcohol misuse during work hours and the risks involved with increased, excessive alcohol consumption. In cases where there are reasons to suspect work-related alcohol use, regular and frequent use of video conferences may be one way of following up employees.

Due to some methodological limitations, the findings should be interpreted with caution. Because all measures were based on self-report, the results may be affected by response set tendencies. For instance, it is possible that some misclassification of alcohol intake may have influenced the findings as underreporting of alcohol use is common in population-based studies and especially among heavy drinkers (Heikkila et al., 2012). The cross-sectional study design limits any conclusions about actual cause-and-effect relationships. That is, with only one survey time point we were only able to determine whether working at home was associated with alcohol use and not whether working at home *leads to* increased employee alcohol use. Altogether 50% of invited respondents did not participate in the questionnaire survey. The external validity of the findings may therefore be questioned. However, nonresponse is a necessary but not sufficient condition for response bias. If the reason for nonresponse is uncorrelated with variables being analyzed, response rates well below 100% do not indicate response bias and lack of generalizability (Groves et al., 2004). Furthermore, as respondents were informed that the survey was completely anonymous, it is unlikely that those with problematic drinking habits has refused to participate due to a fear of having their problems exposed to the employer.

Despite these limitations, this study has established working at home as a potential risk factor for alcohol use. Upcoming research should replicate and extend the study by using prospective designs that allow for examining whether transitioning from on-site work to working at home leads to changes in alcohol consumption. Furthermore, future research should apply methods that assess alcohol use in terms of its temporal relation to the workday (Frone, 2016). To better understand the association between working at home and alcohol use, upcoming research should focus on the causes of problematic drinking, including the motivation for drinking. Factors such as coping strategies, loneliness and lack of social support may be especially important candidates for examination (Bramness et al., 2021).

## Declaration of Competing Interest

The authors declare that they have no known competing financial interests or personal relationships that could have appeared to influence the work reported in this paper.
